# L’épilepsie post-accident vasculaire cérébral: Premières données nigériennes

**DOI:** 10.48327/mtsi.v5i4.2025.654

**Published:** 2025-07-06

**Authors:** Zakaria MAMADOU, Fawzia ABDOULAYE DARA, Moussa TOUDOU DAOUDA, Inoussa DAOUDA BAKO, Amadou ABDOU BACHAROU, Fataoulaye SOUMANA HASSANE, Éric ADEHOSSI

**Affiliations:** 1 Unité neuro vasculaire du service de neurologie de l’hôpital général de référence de Niamey, Niger; 2 Service de neurologie de l’hôpital Amirou Boubacar Diallo de Niamey, Niger; 3 Service d’imagerie médicale de l’hôpital général de référence de Niamey, Niger; 4 Service de médecine interne de l’hôpital général de référence de Niamey, Niger

**Keywords:** Épilepsie post-AVC, Épidémiologie, Score SeLECT, Score CAVE, Niger, Afrique subsaharienne, Post-stroke epilepsy, Epidemiology, SeLECT score, CAVE score, Niger, Sub-Saharan Africa

## Abstract

**Introduction:**

L’épilepsie post-accident vasculaire cérébral (AVC) se définit par la survenue de crises non provoquées chez un sujet aux antécédents d’AVC, après exclusion de toute autre cause.

**Objectif:**

L’objectif de ce travail était de déterminer les caractéristiques épidémio-cliniques, radiologiques et évolutives des épilepsies post-AVC au service de neurologie de l’hôpital général de référence de Niamey (Niger). Il s’agissait d’une étude rétrospective, descriptive et analytique d’une durée de 29 mois.

**Résultat:**

Au total, 4 765 patients ont fait l’objet de cette étude parmi lesquels 21 ont été diagnostiqués épileptiques post-AVC, soit une prévalence hospitalière de 4, 4%. Le sex-ratio était de 0,75. La moyenne d’âge était de 64. LAVC ischémique (AVCI) était majoritaire dans 81% des cas avec des lésions le plus souvent dans le territoire superficiel de l’artère cérébrale moyenne. Les crises étaient le plus souvent focales dans 52,4% des cas, survenant tardivement dans l’intervalle de 1 à 2 ans. La majorité des patients était mise sous monothérapie avec une prédominance de la carbamazépine.

**Conclusion:**

L’évolution était favorable dans 71,5% des cas sous traitement bien conduit.

## Abstract (en)

**Introduction:**

Post-stroke epilepsy is defined as unprovoked seizures occurring in a person with a history of stroke after all other causes have been excluded.

**Objective:**

This study aimed to determine the epidemiological, clinical, radiological, and evolutionary characteristics of post-stroke epilepsy in the neurology department of the general referral hospital in Niamey, Niger. The study was retrospective, descriptive, and analytical, and it was conducted over a period of 29 months. Results. A total of 4,765 patients were included in the study, 21 of whom were diagnosed with post-stroke epilepsy. This represents a hospital prevalence of 4.4%o. The sex ratio was 0.75. The average age was 64. Ischemic stroke (IS) was the most common type, accounting for 81% of cases, with lesions most often occurring in the superficial territory of the middle cerebral artery. Seizures were focal in 52.4% of cases and occurred late, within one to two years. Most patients were treated with monotherapy, primarily with carbamazepine.

**Conclusion:**

The outcome was favorable in 71.5% of cases with appropriate treatment.

## Introduction

L’épilepsie post-accident vasculaire cérébral (EP-AVC) se définit par la survenue de crises non provoquées chez un sujet aux antécédents d’AVC, après exclusion de toute autre cause [3]. Les lésions vasculaires cérébrales exposent à un risque accru de crise épileptique et d’épilepsie. La fréquence des crises épileptiques après un AVC est d’environ 10%; une minorité de ces patients développe une véritable EP-AVC [8]. Il faut aussi préciser que beaucoup de cas d’épilepsie en général sont sous diagnostiqués en Afrique, car associés à des croyances erronées et stigmatisantes malgré l’évolution de la science médicale [2].

Selon *l’International League Against Epilepsy* (ILAE), les crises post-AVC sont classées en crises précoces (dans les 7 jours suivant l’AVC) et en crises tardives (après la première semaine de l’AVC) [12]. Les études d’incidence de l’épilepsie après un AVC en Afrique subsaharienne sont rares étant donné les difficultés de suivi à long terme et de réalisation d’études de cohortes dans cette région [1].

Au Niger, à notre connaissance, aucune recherche n’a été faite sur ce sujet, d’où l’intérêt de notre étude. L’objectif de ce travail a été de présenter les premières données de l’EP-AVC.

## Méthodologie

Le service de neurologie de l’hôpital général de référence de Niamey a été notre site d’étude. Une étude rétrospective, descriptive et analytique a été conduite sur une période de 29 mois (du 1^er^ mai 2021 au 31 octobre 2023). Ont été inclus dans l’étude, tous les patients:

- âgés de 18 ans ou plus sans distinction de sexe,
- admis dans le service de neurologie ou reçus en consultation,
- disposant d’un dossier médical exploitable,
- chez lesquels le diagnostic d’EP-AVC été retenu:
- quels que soient le type d’AVC et le type de crise épileptique révélatrice;
- et selon les critères de l’ILAE.

Le dossier médical du patient a été la source de collecte de nos données: données sociodémographiques; facteurs de risque cardiovasculaire; type d’AVC; scores de prédilection de la survenue d’une EP-AVC: score SeLECT [5] pour les AVC ischémiques (AVCI), score CAVE [7] pour les AVC hémorragiques (AVCH); délai entre l’AVC et l’épilepsie; type des crises; données de l’électroencéphalogramme (EEG); médicament antiépileptique (MAE); évolution clinique sous traitement.

Notre étude a suivi les principes de la déclaration d’Helsinki, et l’approbation d’un comité d’éthique n’a pas été nécessaire. Tous les patients ont donné leur consentement éclairé pour participer à cette étude.

## Résultats

### Caractéristiques sociodémographiques

Au total, sur 4 765 dossiers retenus, 21 cas d’EP-AVC ont été diagnostiqués, soit une prévalence hospitalière totale de 4,4%. De façon plus spécifique, 703 dossiers ont été exploités en hospitalisation avec 15 cas d’EP-AVC (soit une prévalence hospitalière de 2,13%) et 4 062 dossiers en consultation externe avec 6 cas d’EP-AVC (soit une prévalence hospitalière de 1,5%). Le sexratio était de 0,75. L’âge moyen était de 63,7 ans. L’hypertension artérielle (HTA) et le diabète de type 2 étaient les facteurs de risque cardiovasculaire les plus fréquents. Le Tableau I résume les caractéristiques sociodémographiques et les facteurs de risque cardiovasculaire des patients.

**Tableau 1:** Données socio-démographiques, facteurs de risque cardiovasculaire et données de l’imagerie cérébrale dans l’EP-AVC au Niger

Variables	Nombre	Fréquence (%)
Sexe		
homme	9	43
femme	12	57
Âge (ans)		
40 – 50	4	19
51 – 60	5	23,9
61 – 70	3	14,3
71 – 80	7	33,3
> 80	2	9,5
Facteurs de risque cardio vasculaire		
HTA	17	81
diabète	7	33,3
dyslipidémie	10	47,6
sédentarité	20	95,2
tabac	2	9,5
Type de crise d’épilepsie		
crise généralisée	8	38,1
crise focale	11	52,4
crise focale secondairement généralisée	2	9,5
Données de l’imagerie cérébrale		
Territoire AVCI (n = 17)		
ACM superficielle	13	76,4
ACM superficielle et profonde	2	11,8
ACA superficielle	2	11,8
Localisation AVCH (n = 4)		
lombaire	2	50
profonde	1	25
avec inondation ventriculaire/ with ventricular flooding	1	25

HTA: hypertension artérielle; AV CI: accident vasculaire cérébral ischémique; AV CH: accident vasculaire cérébral hémorragique; ACM: artère cérébrale moyenne, ACA: artère cérébrale antérieure

HBP: high blood pressure; IS: ischemic stroke; HS: hemorrhagic stroke; MCA: middle cerebral artery, ACA: anterior cerebral artery

### Caractéristiques cliniques, radiologiques et neurophysiologiques.

L’AVC ischémique (AVCI) était majoritaire dans 81% des cas. Les crises étaient de survenue tardive après l’AVC chez 90,5% des patients. La moyenne du score SeLECT était de 4,2. Le territoire superficiel de l’artère cérébrale moyenne était le plus atteint dans 76,5% des AVCI. La moyenne du score CAVE était de 1,25 et plus de 50% des patients avaient un hématome lobaire. Il s’agissait de crises focales chez 11 patients soit 52,4% des cas (parmi ceux-ci 9 dans le groupe AVCI), et de crises d’emblée généralisées chez 8 patients soit 38,1% des cas (dont 6 dans le groupe d’AVCI). L’EEG était réalisé chez 13 patients, normal pour 10 d’entre eux et pathologique pour 3. Les anomalies épileptiques étaient focales chez deux patients et généralisées chez un seul. L’EEG était pathologique chez deux patients AVCI.

### Évolution sous traitement

Les patients étaient suivis en consultation régulière. Aucun d’entre eux n’a été perdu de vue. Les crises d’épilepsie étaient contrôlées par un seul MAE de fond chez 17 patients (soit 81% des cas), alors que chez 4 patients (19% des cas) il a été associé à une benzodiazépine. La carbamazépine a été le MAE de fond le plus prescrit chez 38,1% des patients.

## Discussion

La prévalence hospitalière de l’EP-AVC dans notre étude était de 2,13%. La fréquence de l’EP-AVC dans la population générale reste rare en Afrique subsaharienne. Il existe cependant quelques études hospitalières menées avec des méthodologies différentes. Ainsi la prévalence hospitalière est estimée à 2% à Cotonou (Bénin) [6] ainsi qu’à Ouagadougou (Burkina Faso) [10]. Ces résultats sont similaires à ceux de notre étude. L’âge moyen de notre population d’étude était de 63,7 ans, nettement supérieur à celui de Gnonlonfoun [6] (53 ans) mais proche de l’âge moyen trouvé dans l’étude de Napon et collaborateurs [10] qui était de 58 ans. En effet, l’épilepsie du sujet âgé est cinq à sept fois plus fréquente que celle du sujet jeune, suite principalement à des pathologies neurovasculaires dont les AVC en premier lieu. La prédominance des épilepsies post-AVCI s’explique par la fréquence de ce type d’AVC dans le monde [4,9]. Dans notre série, l’AVCI représentait 81% des cas. Cette observation a été rapportée dans plusieurs autres études en Afrique subsaharienne [6,11]. Dans notre étude, 54% des patients avaient un score SeLECT à 5 dans les AVCI et 50% de nos patients une valeur à 2 pour le score CAVE dans les AVCH. L’utilisation de ces scores n’a pas beaucoup d’intérêt dans notre étude car il s’agissait des scores prédictifs de survenue d’épilepsie après AVC, qui tenaient compte des différents facteurs de risque. Néanmoins, ils nous ont servi à faire ressortir que la majorité des lésions se retrouvait dans le territoire superficiel de l’artère cérébrale moyenne dans près de 76,5% des cas pour les AVCI, et avec une localisation lobaire des hématomes dans 50% des cas pour les AVCH.

## Conclusion

Les EP-AVC sont dominées par des crises focales et les AVCI constituent la forme d’AVC la plus épileptogène. Les MAE de première génération gardent encore leur place dans l’arsenal thérapeutique et la carbamazépine était le plus prescrit dans notre étude avec un bon contrôle des crises épileptiques.

## Financement de l’étude

Cette étude n’a reçu aucun financement.

## Contribution des auteurs

Conception et réalisation de l’étude: ZM et FAD. Collecte des données: ZM, FAD et FSH. Analyse et interprétation des données: ZM, MTD, AAB, IDB et EA. Recherche documentaire: ZM, MTD, IDB. Rédaction du manuscrit: ZM et FAD. Révision du manuscrit: ZM, FAD, FSH, IDB, MTD et EA. Garant de l’étude: ZM.

## Conflits d’intérêts

Les auteurs ne déclarent aucun conflit d’intérêts.
