# Supplementary material for: Post-stroke epilepsy: First data from Niger
Source: Med Trop Sante Int. 2025 Jul 6;5(4):mtsi.v5i4.2025.654. [Article in French] doi: 10.48327/mtsi.v5i4.2025.654 (PMC12948368; doi:10.48327/mtsi.v5i4.2025.654)
Supplement: Related texts

# Research article: Post-stroke epilepsy: First data from Niger

## Introduction

Post-stroke epilepsy (PSE) is defined as unprovoked seizures occurring in a person with a history of stroke after all other causes have been excluded [3]. Cerebral vascular lesions increase the risk of epileptic seizures and epilepsy. Approximately 10% of people experience epileptic seizures after a stroke, and a minority of these patients develop true PSE [8]. It should also be pointed out that many cases of epilepsy in general are under-diagnosed in Africa, because they are associated with erroneous and stigmatizing beliefs, despite advances in medical science [2].

The International League Against Epilepsy (ILAE) classifies post-stroke seizures as early (within seven days of the stroke) or late (after the first week of the stroke) [12]. Studies on the incidence of epilepsy after stroke in sub-Saharan Africa are rare, given the difficulty of conducting long-term follow-ups and cohort studies in this region [1]. To our knowledge, no research has been conducted on this topic in Niger; hence, the importance of our study. The objective of this study was to present the first data on PSE.

## Methodology

The neurology department of the Niamey General Reference Hospital served as the study site. We conducted a retrospective, descriptive, and analytical study over a 29-month period (from May 1, 2021, to October 31, 2023). Were included in the study all patients:

- aged 18 years or older, regardless of gender,
- admitted to the neurology department or seen in consultation,
- with a usable medical record,
- in whom a diagnosis of PSE was made:
- regardless of the type of stroke and the type of epileptic seizure;
- and according to the ILAE criteria.

We collected data from the patients’ case report form, including sociodemographic data, cardiovascular risk factors, type of stroke, and scores for the likelihood of developing PSE. The SeLECT score [5] was used for ischemic strokes (IS) and the CAVE score [7] for hemorrhagic strokes (HS). We also considered the time between the stroke and the onset of epilepsy, the type of seizures, electroencephalogram (EEG) data, antiepileptic drugs (AEDs), and clinical progression under treatment.

Our study adhered to the principles of the Declaration of Helsinki, and ethics committee approval was not required. All patients provided informed consent to participate in the study.

## Results

### Sociodemographic characteristics

Out of the 4,765 selected records, 21 cases of PSE were diagnosed, representing a hospital prevalence of 4.4 per 1,000. Specifically, 703 records from hospital admissions were analyzed, yielding 15 cases of PSE (a prevalence of 2.13%), and 4,062 outpatient consultation records were analyzed, yielding six cases of PSE (a prevalence of 1.5%o). The sex ratio was 0.75. The average age was 63.7 years. High blood pressure (HBP) and type 2 diabetes were the most prevalent cardiovascular risk factors. Table I summarizes the sociodemographic characteristics and cardiovascular risk factors of the patients.

**Table 1:** Socio-demographic data, cardiovascular risk factors, and brain imaging data in PSE in Niger

Variables	Number	Frequency%
Gender		
male	9	43
female	12	57
Age (years)		
40 – 50	4	19
51 – 60	5	23,9
61 – 70	3	14,3
71 – 80	7	33,3
> 80	2	9,5
Cardiovascular risk factors		
HBP	17	81
diabetes	7	33,3
dyslipidemia	10	47,6
sedentary lifestyle	20	95,2
smoking	2	9,5
Type of epileptic seizure		
generalized seizure	8	38,1
focal seizure	11	52,4
secondary generalized focal seizure	2	9,5
Brain imaging data		
IS region (n = 17)		
superficial MCA	13	76,4
superficial and deep MCA	2	11,8
superficial ACA	2	11,8
HS location (n = 4)		
lumbar	2	50
deep	1	25
avec inondation ventriculaire/ with ventricular flooding	1	25

HTA: hypertension artérielle; AV CI: accident vasculaire cérébral ischémique; AV CH: accident vasculaire cérébral hémorragique; ACM: artère cérébrale moyenne, ACA: artère cérébrale antérieure

HBP: high blood pressure; IS: ischemic stroke; HS: hemorrhagic stroke; MCA: middle cerebral artery, ACA: anterior cerebral artery

### Clinical, radiological, and neurophysiological characteristics

Ischemic stroke (IS) was the most common type, accounting for 81% of cases. Symptom onset was delayed in 90.5% of patients. The mean SeLECT score was 4.2. The superficial territory of the middle cerebral artery was most affected in 76.5% of ISs. The mean CAVE score was 1.25, and over 50% of patients had a lobar hematoma. Focal seizures occurred in 11 patients (52.4% of cases), including 9 in the IS group. Generalized seizures occurred in 8 patients (38.1% of cases), including 6 in the IS group. EEGs were performed on 13 patients: 10 were normal and 3 were abnormal. Epileptic abnormalities were focal in two patients and generalized in only one. Two IS patients had abnormal EEGs.

### Progression under treatment

Patients were monitored during regular consultations. None were lost to follow-up. A single long-term AED controlled epileptic seizures in 17 patients (81% of cases), while a benzodiazepine was combined with an AED in four patients (19% of cases). Carbamazepine was the most commonly prescribed long-term AED, prescribed to 38.1% of patients.

## Discussion

The hospital prevalence of PSE in our study was 2.13%. PSE remains rare in the general population of sub-Saharan Africa. However, only a few hospital studies have been conducted using different methodologies. Thus, the hospital prevalence is estimated at 2% in Cotonou, Benin [6], and Ouagadougou, Burkina Faso [10]. These results are similar to those of our study. The average age of our study population was 63.7 years, which is significantly higher than the average age of 53 years found in the Gnonlonfoun study [6], but close to the average age of 58 years found in the Napon et al. study [10]. Epilepsy is five to seven times more prevalent in older adults than in younger adults, primarily due to neurovascular conditions, particularly stroke. Post-stroke epilepsy is prevalent because stroke is common worldwide [4,9]. In our series, IS accounts for 81% of cases. This observation has been reported in several other studies in sub-Saharan Africa [6,11]. In our study, 54% of patients had a SeLECT score of 5 in IS and 50% of our patients had a CAVE score of 2 in HS. These scores are not particularly relevant to our study because they predict the onset of epilepsy following a stroke taking various risk factors into account. Nevertheless, these scores helped us determine that most lesions were in the superficial territory of the middle cerebral artery, accounting for nearly 76.5% of IS cases, and with a lobar location of hematomas in 50% of cases for HS.

## Conclusion

PSE are dominated by focal seizures, and ISs are the most epileptogenic form of stroke. First-generation AEDs still have a place in the therapeutic arsenal, and carbamazepine was the most commonly prescribed drug in our study, with good control of epileptic seizures.

## Funding

This study received no funding.

## Authors’ contributions

Study design and implementation: ZM and FAD. Data collection: ZM, FAD, and FSH. Data analysis and interpretation: ZM, MTD, AAB, IDB, and EA. Literature review: ZM, MTD, IDB. Manuscript writing: ZM and FAD. Manuscript revision: ZM, FAD, FSH, IDB, MTD, and EA. Study guarantor: ZM.

## Conflicts of interest

The authors declare no conflicts of interest.
